# Building capacity, driving impact: A landscape analysis of community engagement and outreach cores’ activities

**DOI:** 10.1017/cts.2026.10781

**Published:** 2026-07-26

**Authors:** Emily Frankel, Stephanie Broyles, Stephenie Kennedy-Rea, Casey Solsrud, Elizabeth Woods, Deborah Goebert, Zsolt Nagykaldi, Holly Huye, Lee M. Pachter

**Affiliations:** 1 Family Medicine, University of Nebraska Medical Center, Omaha, NE, USA; 2 Great Plains IDeA-CTR, Omaha, NE, USA; 3 Community Health Science & Policy, Louisiana State University Health Sciences Center New Orleans, New Orleans, LA, USA; 4 LA CaTS, Baton Rouge, LA, USA; 5 Cancer Prevention and Control, West Virginia University School of Medicine, Morgantown, WV, USA; 6 WVCTSI, Morgantown, WV, USA; 7 Neurological Sciences, University of Nebraska Medical Center, Omaha, NE, USA; 8 UVM Larner College of Medicine, Burlington, VT, USA; 9 Northern New England Clinical and Translational Research Network, Burlington, VT, USA; 10 Psychiatry, University of Hawai’i at Mānoa John A Burns School of Medicine, Honolulu, HI, USA; 11 Center for Pacific Innovations, Knowledge, and Opportunities, Honolulu, HI, USA; 12 Family and Preventive Medicine, The University of Oklahoma Health Sciences Center, Oklahoma City, OK, USA; 13 Oklahoma Shared Clinical and Translational Resources, Oklahoma City, OK, USA; 14 College of Education and Human Sciences, University of Southern Mississippi, Hattiesburg, MS, USA; 15 Mississippi Center for Clinical and Translational Research, Jackson, MS, USA; 16 Pediatrics & Population Health, Thomas Jefferson University, Philadelphia, PA, USA; 17 Institute for Research on Equity & Community Health, Christiana Care Health Services Inc, Wilmington, DE, USA; 18 Delaware CTR ACCEL Program, Newark, DE, USA

**Keywords:** Community engagement, capacity building, research infrastructure, knowledge translation, learning collaborative

## Abstract

Community Engagement and Outreach (CEO) Cores are a key component of the IDeA Clinical and Translational Research (IDeA-CTR) Program, serving to develop and implement capacity-building initiatives that empower communities to engage and collaborate with investigators in community-engaged research. To strengthen collaboration and shared learning across sites, a CEO Special Interest Group was established. In 2022, the group conducted a landscape analysis to systematically characterize and categorize CEO Core activities across the IDeA-CTR network, with a focus on capacity-building efforts related to knowledge transfer, co-learning, and infrastructure development. The analysis revealed several common approaches to community engagement and capacity building across sites, as well as distinctive strategies tailored to the specific needs and communities served. Multiple innovative practices were identified and disseminated across the network, highlighting opportunities to enhance collective impact and inform future community-engaged efforts.

## Context: the IDeA-CTR and community engagement and outreach cores

The National Institute of General Medical Sciences (NIGMS) established the Institutional Development Award (IDeA) in 1993 as a Congressional Mandate to broaden the distribution of National Institutes of Health (NIH) funding for biomedical and behavioral research in states that historically receive limited NIH funding [1,2]. The IDeA Program includes the IDeA Clinical and Translational Research Program (IDeA-CTR), which focuses on enhancing and strengthening research infrastructure and human resources to advance clinical and translational research (CTR) that responds to the needs of medically and geographically under-resourced communities [2]. Advancing CTR to address population health challenges requires strong collaboration and partnership between researchers and community members, anchored in trust and engagement [3–5]. Consequently, Community Engagement and Outreach (CEO) Cores are mandated as part of the infrastructure within each funded IDeA-CTR program [6].

CEO Cores support multi-directional engagement, partnership, and reciprocity between CTR and community members by integrating communities throughout the research process. Through these efforts, CEO Cores ensure community priorities and health concerns are prioritized and community perspective and lived experiences are honored. To foster and sustain meaningful collaboration and research partnership, CEO Cores develop and implement capacity-building initiatives and activities that empower communities to engage and collaborate with IDeA-CTR investigators on community-engaged research (CEnR) initiatives. CEnR readiness, for both community members and researchers, requires individual competencies (e.g., knowledge, skills, frameworks, and leadership), organizational infrastructure (e.g., culture, policies, incentives, and governance), and societal support (e.g., collaboration across organizations and sectors) [7–9]. Building individual, organizational, and societal capacity ultimately strengthens CEnR impact by enhancing the ability of community partners and researchers to co-develop, plan, implement, evaluate, and disseminate research that improves health outcomes and informs real-world practice [6,8].

In an effort to strengthen the impact of IDeA-CTR CEO Cores, a CEO Special Interest Group (SIG) was established in September 2022 to facilitate cross-CTR learning, collaboration, and the advancement of CEnR. The CEO SIG is comprised of 26 CEO Core Directors, Co-Directors, and staff, representing 12 IDeA-CTR networks (Table 1). The CEO SIG serves as a community of practice to promote knowledge sharing and peer learning, networking, relationship building, and dissemination of best practices to achieve the overall goals of the CEO Cores. While the guiding principles and CEO Core directives remain constant across the IDeA-CTR networks, the individual Cores often achieve these goals through a range of community engagement (CE) activities. In this paper, we summarize the heterogeneity in CEO Core capacity-building activities across IDeA-CTR programs. Additionally, we highlight innovative initiatives through which CEO Cores tailor programming to meet the unique needs of their community members and CTR investigators, while also identifying gaps where activities could be enhanced to generate greater impact.

**Table 1.** Participating IDeA-CTR networks in the CEO SIG

IDeA-CTR network	State(s)	Number of key personnel in CEO SIG
Accelerating Clinical and Translational Research (ACCEL)	Delaware	6
Advance Rhode Island Clinical and Translational Research (Advance)	Rhode Island	1
Hispanic Alliance for Clinical & Translational Research (Alliance)	Puerto Rico	2
Great Plains IDeA Clinical and Translational Research (GP IDeA-CTR)	Nebraska	3
Louisiana Clinical & Translational Science Center (LA CaTs)	Louisiana	2
Mississippi Center for Clinical and Translation Research (MCCTR)	Mississippi	3
Mountain West Clinical and Translational Research Infrastructure Network (MW CTR-IN)	Montana, Wyoming, Idaho, Nevada, New Mexico, Alaska, Hawaii	3
Montana Center for Clinical and Translational Research Center (CTRC)	Montana	1
Northern New England (NNE-CTR) Clinical & Translational Research Network	Vermont, New Hampshire, Maine	1
Oklahoma Shared Clinical and Translational Resources (OSCTR)	Oklahoma	1
Center for Pacific Innovations, Knowledge, and Opportunities (PIKO)	Hawaii	2
West Virginia Clinical and Translational Science Institute (WVCTSI)	West Virginia	1

IDeA = institutional development award; CTR = clinical and translational research; CEO = community engagement and outreach; SIG = special interest group.

## Approach: landscape analysis of capacity-building activities

**Figure 1.**
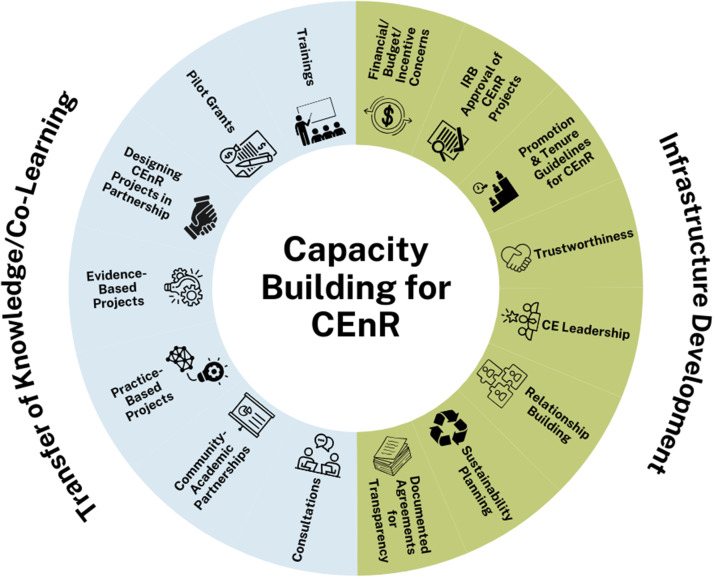
Figure 1 long description. Capacity-building activities for CEnR. CEnR = community-engaged research; IRB = institutional review board; CE = community engagement.

Since September 2022, the CEO SIG has convened quarterly through teleconference meetings. During early meetings, members of the CEO SIG identified a need to conduct a landscape analysis to characterize and systematically document the range of capacity-building initiatives occurring within CEO Cores. Capacity-building activities are those that strengthen skills, systems, structures, and relationships to effectively achieve goals [10]. Capacity building is intended to generate change at various levels of the ecosystem through resource allocation and distribution [11]. To initiate the landscape analysis, a data collection tool was developed and circulated to all CEO Cores in October 2023, collecting the following information for each capacity-building activity: (1) Context, (2) Time, (3) Location, (4) Platform (in-person/virtual), (5) Number of participants, (6) Participant demographics, (7) Curriculum/content, (8) Focus/area of emphasis, and (9) Partnerships/collaborators. Concurrently, a deductive approach was used to establish preliminary codes from a comprehensive literature review to identify the essential capacity-building elements in CEnR [12]. Between November 2023 and April 2024, the CEO SIG used meetings to facilitate data collection through iterative facilitated discussions and reviewed capacity-building elements identified in the literature. A classification scheme was then developed according to the themes that emerged from the literature, which were evaluated and adapted to ensure alignment with CE and CEnR principles. The classification scheme included two broad domains: (1) Transfer of Knowledge/Co-Learning and (2) Infrastructure Development [7,10,13–18]. The list of themes, including brief descriptions, was presented to the CEO SIG to confirm face validity and achieve consensus, ensuring clarity and agreement on the thematic framework (Figure 1 and Table 2).

**Table 2.** CEnR capacity-building domains, themes, and their descriptions

Thematic areas			
Transfer of knowledge/co-learning		Infrastructure development	
Themes	Description	Themes	Description
Trainings	Learning opportunities in the form of seminars, webinars, workshops, institutes, etc., that build skills for CEnR	Financial/Budget/Incentive Concerns	Developing fair and transparent policies for budgetary considerations, community compensation, resource allocation, and sustainability strategies
Pilot grants	Funding opportunities to build teams, test feasibility, collect pilot data, or strengthen partnerships to advance CEnR	IRB Approval of CEnR Projects	Ensuring the IRB understands and honors principles of community engagement when reviewing applications, demonstrating institutional infrastructure for CEnR
Designing CEnR projects in partnership	A process where researchers and community partners co-create a research project to ensure relevance and benefit to the community	Promotion & Tenure Guidelines for CEnR	Establishing guidelines for promotion and tenure that acknowledge and reward community engagement activities for academic career advancement
Evidence-based projects	Through the design, adaptation, implementation, and evaluation of an evidence-based project, skills and confidence of researchers and community partners are strengthened	Trustworthiness	Demonstrating and improving trustworthiness to strengthen research systems for effective collaboration
Practice-based projects	Identifying real-world, clinical needs and empowering practitioners, patients, and researchers to collaborate to address such needs	CE Leadership	Cultivating leaders within academic settings who champion and guide the implementation of CEnR principles
Community-academic partnerships	Ongoing, mutually beneficial relationships between community members and academic researchers in the pursuit of CEnR	Relationship Building	Honoring that relationships with the community create the foundation on which CEnR projects develop and create formal structures for engagement, such as advisory boards or coalitions
Consultations	Individual or group meetings with community-engaged scholars or experts to inform community engagement in translational research	Sustainability Planning	Developing strategies and structures to support community-academic partnerships beyond the life of a specific grant or funding opportunity
		Documented Agreements for Transparency	Generating agreements such as Memorandums of Understanding and partnership charters that outline roles and responsibilities for transparency and accountability

CEnR = Community-Engaged Research; IRB = Institutional Review Board; CE = Community Engagement.

Six of the 26 CEO SIG members formed a project analysis group, meeting monthly from April 2024 and December 2024, to translate the CEO SIG’s ideas and discussions into documented projects and deliverables, ensuring structured progress and clear communication outcomes. The project analysis group used a team-based coding approach to systemically aggregate and classify the reported capacity-building activities within the a priori themes presented in Table 2. If the capacity-building activity did not align with one of the a priori themes, it was assigned to one of two new themes, “Knowledge Transfer: Other” or “Infrastructure: Other.” Each activity could be assigned to more than one capacity-building domain and theme. The thematic classifications were returned to the broader CEO SIG to ensure validity and accuracy.

**Figure 2.**
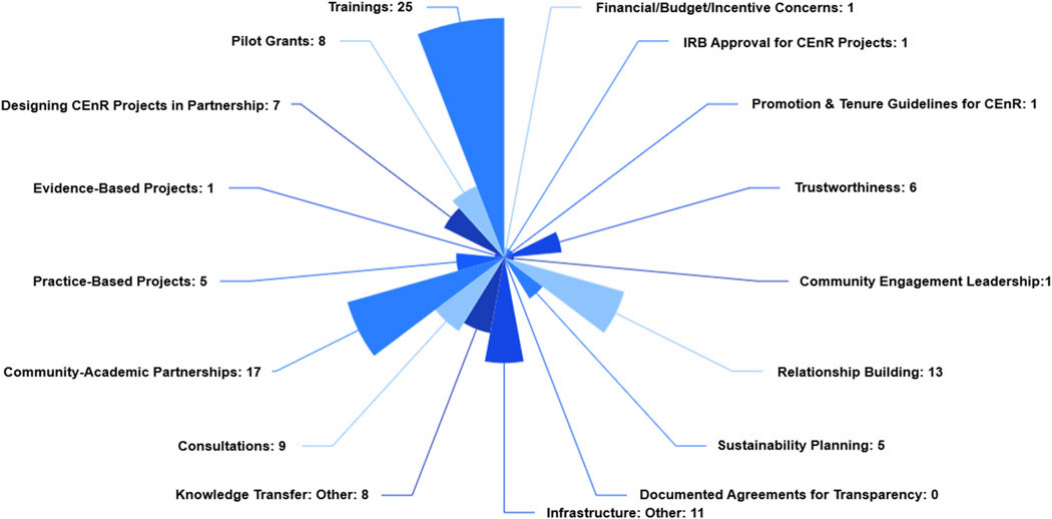
Nightingale Rose Chart of frequency of capacity-building activities. Activities included trainings (*n* = 25), pilot grants (*n* = 8), designing community-engaged research (CEnR) projects in partnership (*n* = 7), evidence-based projects (*n* = 1), practice-based projects (*n* = 5), community-academic partnerships (*n* = 17), consultations (*n* = 9), knowledge transfer: other (*n* = 8), financial/budget/incentive concerns (*n* = 1), institutional review board (IRB) approval for CEnR projects (*n* = 1), promotion and tenure guidelines for CEnR (*n* = 1), trustworthiness (*n* = 6), community engagement leadership (*n* = 1), relationship building (*n* = 13), sustainability planning (*n* = 5), documented agreements for transparency (*n* = 0), and infrastructure: other (*n* = 11).

The project analysis group integrated the data collection tool and thematic classification into a single deliverable (Appendix A), organizing activities by (1) Activity name, (2) Capacity-building theme(s), (3) Audience, and (4) Objectives. Descriptive statistics were used to summarize activities across the a priori themes. Since activities could be assigned to more than one capacity-building theme, the sum of percentages exceeded 100%. A Nightingale Rose Chart was generated using the web platform, Agents for Data, to illustrate categorical variation and the magnitude of activities corresponding to the capacity-building themes (Figure 2).

## Findings: summary of capacity-building activities

### Domains and themes

An analysis of the literature on capacity-building elements for CEnR yielded several themes that were grouped into two thematic areas: (1) Transfer of Knowledge/Co-Learning and (2) Infrastructure Development [7,10,13–18]. Within the thematic area of Transfer of Knowledge/Co-Learning, the following themes emerged: (1) Trainings, (2) Pilot grants, (3) Designing CEnR projects in partnership, (4) Evidence-based projects, (5) Practice-based projects, (6) Community-academic partnerships, and (7) Consultations, reflecting how knowledge is shared and applied. Infrastructure Development was characterized by the following themes: (1) Financial/budget/incentive concerns, (2) IRB approval for CEnR projects, (3) Promotion and tenure (P&T) guidelines for CEnR, (4) Trustworthiness, (5) Community engagement leadership, (6) Relationship building, (7) Sustainability planning, and (8) Documented agreements for transparency, highlighting systems and resources to support CEnR.

### Capacity-building themes

A total of 64 unique capacity-building activities were offered across the 12 IDeA-CTR networks. Many activities (*N* = 25) spanned multiple capacity-building themes, some of which (*N* = 15) were dually classified as Transfer of Knowledge/Co-Learning and Infrastructure Development, accounting for a total of 119 thematic classifications. Transfer of Knowledge/Co-Learning activities were most frequently reported (*N* = 80, 67.2%). Trainings (*N* = 25, 31.3%) and community-academic partnerships (*N* = 17, 21.3%) emerged as the most common Transfer of Knowledge/Co-Learning activities, demonstrating a priority for strengthening practical skills and promoting cross-sector partnerships. Fewer Transfer of Knowledge/Co-Learning activities focused on research projects, such as practice-based projects (*N* = 5, 6.3%) and evidence-based projects (*N* = 1, 1.3%), demonstrating an emphasis on strengthening foundational capacity necessary to support and sustain CEnR projects and engagement (Figure 2). Infrastructure Development activities were less frequently reported (*N* = 39, 32.8%), with relationship building occurring at the greatest frequency (*N* = 13, 33.3%). The emphasis on relationship-building activities underscores the importance of collaboration and partnership in CEnR. In contrast, systems-level and organizational infrastructure, financial/budget/incentive concerns (*N* = 1, 2.6%), IRB approval for CEnR projects (*N* = 1, 2.6%), P&T guidelines for CEnR (*N* = 1, 2.6%), community engagement leadership (*N* = 1, 2.6%), and documented agreements for transparency (*N* = 0, 0%), were reported less often (Figure 2).

### Audience

Across the CEO Core activities, capacity-building activities reached a broad representation of key partners, including junior and senior investigators, community members, clinicians, public health professionals, students and trainees, patients, community engagement leadership, and staff. Most activities were geared towards diverse audiences, representing two or more key partner groups (*N* = 43, 67.2%). A majority of activities incorporated community members and patients as key audience members (*N* = 47, 73.4%).

### Objectives

The objectives of capacity-building activities ranged in breadth and depth. Some activities had specific objectives (e.g., administer community-engaged pilot awards, build a customer relationship management database, and establish community-driven health priorities), while other activities had broad objectives (e.g., training on CEnR, provide support for CEnR, build relationships, and mentoring). The majority of objectives were oriented towards building individual capacity (*N* = 43, 67.2%), while some focused on societal capacity (*N* = 19, 29.7%), and few addressed organizational capacity (*N* = 2, 3.1%).

## Implications: strengths, innovations, and opportunities for growth

This landscape analysis of CEO Core capacity-building activities assessed the range of activities implemented across IDeA-CTR networks. Findings from this analysis revealed heterogeneity in activity scope, intended audience, and objectives. While CEO Cores share a common goal to develop and implement capacity-building initiatives to advance CEnR, approaches vary in their focus, delivery method, duration, target audience, and intended outcomes. The range of capacity-building activities reflects the flexibility of the IDeA-CTR program to tailor activities to each networks’ unique communities, partner needs and priorities, network resources, available infrastructure, and existing societal capacity.

The majority of capacity-building activities focused on individual capacity through trainings, emphasizing the networks’ shared commitment to enhancing research skills, and fostering collaborative learning. Activities such as West Virginia’s ECHOs and design studios, Northern New England’s Boot Camp Translation Training, and Hawaii’s Institute showcase novel training platforms that increase knowledge, build confidence, and enhance peer support. A similar emphasis on trainings have been observed in prior literature examining capacity development, emphasizing the necessity and importance of knowledge and skills acquisition to effectively conduct meaningful CEnR [7,13,17,19]. Individual capacity is essential for building competence to effectively engage in CEnR, often resulting in increased trust, research literacy, and mutual understanding [7,18,20,21].

Other frequently reported capacity-building activities emphasized enabling community-academic partnerships and relationship building. Both activities encompass individual, organizational, and societal capacity, serving as the linkage between all three layers. At the individual level, community-academic partnerships and relationship building are rooted in trust, enhance communication, strengthen cultural humility, and require shared decision making [7,18,20,21]. At the organization level, partnerships and relationships require formalized commitments and agreements between universities, health departments, and community-based organizations with a shared governance structure [22]. Community-academic partnerships and relationships are generally cross-sector in nature and aim to address multi-faceted community priorities, demonstrating societal-level partnership capacity [23,24]. In the scope of CTR, these partnerships and relationships are often derived from Practice-Based Research Networks (Rhode Island, Montana, Mountain West region, Hawaii, and Oklahoma), Community Advisory Boards (Delaware, Rhode Island, Nebraska, Northern New England, Hawaii, and West Virgina), and partnership programs (Delaware, Puerto Rico, Nebraska, Mountain West region, and West Virgina).

Innovative capacity-building activities unique to CEO Cores emerged, demonstrating flexibility, responsiveness, and commitment to addressing community-identified priorities. Of note, Delaware’s Junior Investigator Network (JIN) focuses on CEnR mentorship and training for early-career investigators, who may otherwise face challenges or barriers navigating the research ecosystem. Rhode Island launched a community engagement database, mimicking a customer relations management system to align organizational resources with community needs and activities. Louisiana developed a Community Research for Optimal Wellness Network (CROWN) to close the research loop by disseminating CEnR outputs back to communities of interest. Mississippi developed CEnR e-modules, allowing asynchronous, continuous learning reaching a broad audience. Together, these activities highlight the IDeA-CTR and CEO Cores’ ability to adapt, evolve, and respond to the needs of network and community members.

This landscape analysis also revealed areas of opportunity and growth for CEO Core activities. Across all CEO Cores, there were no activities geared towards agreements for transparency, such as memorandums of understanding, business associate agreements, or other partnership contracts, a critical component of CEnR for defining partnerships, structure, and governance [22]. Only one CEO Core worked with university and CE leadership to address P&T guidelines for CEnR. Nebraska’s CEO Core intentionally convened CE leadership to discuss revisions of P&T guidelines to include CE and CEnR as an “Area of Review for Health Professions and Special Appointments,” to accompany teaching, research, and service. The limited number of capacity-building activities at the organizational level aligns with previous literature, emphasizing the persistent gap in institutional recognition and support of CEnR [25,26].

To increase sustainable, impactful CEnR, there must be a concerted effort to enhance organizational capacity [18]. We can leverage proven models for adaptation and implementation, such as Cedars-Sinai Cancer Center’s Certificate in Community Outreach and Engagement Training Program, which standardizes education and learning for early-career staff to enable a skilled workforce [27]. The Advancing Research Impact in Society (ARIS) Organizational Research Impact Capacity (ORIC) Program offers another model for increasing organizational capacity by building skills, systems, and processes within an institution to communicate research impact. Through a community of practice, the ARIS ORIC promotes faculty development, research communication, and funding models for sustainability [28].

Prior studies have demonstrated how organizational capacity and infrastructure foster sustainability, enhance efficiency, and promote engagement [8,18,19]. Organizational capacity is anchored in structures, policies, and intra-institutional culture, which are essential for aligning individual motivation with societal commitment [7,13,17]. Insufficient organizational capacity renders engagement activities superficial, academic focus hampers sustainable community relationships, and power imbalances diminish community voices, undermining long-term impact and genuine participation [29,30]. Strengthening organizational capacity would promote and recognize community engagement efforts, reflected in strategic plans, promotion requirements, and internal funding opportunities. Furthermore, organizational capacity would lead to sustainable partnerships and collaborations anchored on shared missions and goals, rather than individual grants or funding opportunities [7,13,17].

While this landscape analysis generated robust insight into CEO Core activities, notable challenges and limitations should be acknowledged. Due to the lack of standardized tracking and evaluation across IDeA-CTR activities, data collection relied on self-reports from members of the CEO SIG who were active participants in the quarterly meetings. This resulted in variable data quality, incomplete data, and inconsistency in reporting. The project analysis group was organized to overcome some of these limitations, but without historical knowledge of each activity, limitations arose. Additionally, due to the composition of IDeA-CTR networks, capacity-building activities may be housed and led by other cores, such as the Professional Development Core, resulting in missing data due to cross-core collaborations. CEO Cores are constantly innovating and implementing new activities, so this landscape analysis provides a snapshot of the activities administered between October 2023 and April 2024. Finally, the IDeA-CTR networks are not on the same funding cycles, leading to subtle nuances in program requirements and expectations. Despite these limitations, this landscape analysis offers valuable insight into the uniqueness of capacity-building activities across CEO Cores and highlights opportunities for growth, enhancement, and collaboration to maximize the impact of activities.

Future work should prioritize standardized reporting, metrics, and evaluation to assess and communicate the reach, effectiveness, outcomes, and sustainability of capacity-building activities across IDeA-CTR networks and the broader CEnR community. Establishing shared metrics will enable comparability and benchmarking across networks, track progress and growth over time, facilitate evidence-based decision-making, promote best practices and shared learning, and aid in identifying gaps in programming that can lead to unmet needs, inefficiencies, and missed opportunities for innovation.

## Supporting information

10.1017/cts.2026.10781.sm001 Frankel et al. supplementary material Frankel et al. supplementary material

## Supplementary material

The supplementary material for this article can be found at https://doi.org/10.1017/cts.2026.10781.

## Figure 1. Long description

This circular infographic is divided into two domains; Transfer of Knowledge/Co-Learning and Infrastructure Development. The Transfer of Knowledge/Co-Learning domain is further divided into 7 themes: 1. trainings, 2. pilot grants, 3. designing CEnR projects in partnership, 4. evidence-based projects, 5. practice-based projects, 6. community-academic partnerships, and 7. consultations. The Infrastructure Development domain is further divided into 8 themes: 1. financial/budget/incentive concerns, 2. IRB approval for CEnR project, 3. promotion and tenure guidelines for CEnR, 4. trustworthiness, 5. CE leadership, 6. relationship building, 7. sustainability planning, and 8. documented agreements for transparency.

Navigate back to Figure 1..
