# Supplementary material for: Building capacity, driving impact: A landscape analysis of community engagement and outreach cores’ activities
Source: J Clin Transl Sci. 2026 Jul 26;10(1):e124. doi: 10.1017/cts.2026.10781 (PMC13420138; doi:10.1017/cts.2026.10781)
Supplement: Frankel et al. supplementary material [file S205986612610781Xsup001.docx]

| *Appendix A. Supplemental table of capacity-building activities across CEO Cores.* | | | | |
| --- | --- | --- | --- | --- |
| ***CTR Name*** | ***Activity*** | ***Capacity-Building Theme(s)*** | ***Audience*** | ***Objectives*** |
| ***ACCEL (Delaware)*** | *Community Engaged Research Consultations* | *Trainings, Pilot Grants, Consultations* | *Investigators* | *Provide support and mentorship for community engagement, community advisory panels, social and structural determinants of health, developing lay descriptions of work, and dissemination* |
|  | *Online lecture series – ACCL Community Engagement (ACE) Curriculum* | *Trainings* | *Community Members, Investigators* | *Training on CEnR* |
|  | *Community Research Exchange (CRE)* | *Trainings, Community-Academic Partnerships, Relationship Building* | *Community Members, Investigators, Clinicians, Students* | *Host a day-long conference on CEnR, including keynote presentations, community panel discussions, research presentations, and workshops* |
|  | *ACCEL Junior Investigator Network (JIN)* | *Trainings, Knowledge Transfer: Other* | *Junior Investigators* | *CTR Professional Development core hosts weekly meetings which include CEnR perspectives from CEO/CAC members* |
|  | *Community Engagement Core (CEO)/Community Advisory Council (CAC) meetings* | *Community-Academic Partnerships, Relationship Building, Infrastructure: Other* | *Community Members, CE Leadership, Investigators* | *Deliver business, informational, and educational content* |
|  | *Community Engagement scoring of CTR pilot proposals* | *Pilot Grants* | *Community Members, CE Leadership* | *Provide community engagement review of CTR pilot proposals regarding strengths and opportunities for improvement in the areas of CEnR specific to the proposal* |
|  | *ACCEL Strategic Initiative Partnership Program (ASIPP)* | *Community-Academic Partnerships, Relationship Building* | *Community Members, Investigators* | *Provide in-kind CTR support to ongoing efforts of community organizations and other entities that have programs aimed at improving the health of Delawareans, provide evaluative, statistical, and community engagement support* |
|  | *Interdisciplinary New Collaboration Award (INC)* | *Designing CEnR Projects in Partnership, Relationship Building* | *Community Members, Investigators* | *Provide funding to develop interdisciplinary teams to address issues that affect the health of Delawareans from a multi/interdisciplinary perspective* |
|  | *Community Engaged Pilot Awards* | *Pilot Grants* | *Investigators* | *Administer Community-Engaged Pilots Awards* |
| ***Advance (Rhode Island)*** | *Consultations* | *Consultations* | *Investigators, Community Members* | *Pilot grant support, Practice-Based Research Network (PBRN) support* |
|  | *Community Engagement Studios* | *Trainings, Community-Academic Partnerships* | *Investigators, Community Members* | *Facilitate the collaborative engagement of community experts and researchers to gather study-specific information that is meaningful to all members of the research team* |
|  | *Community Engaged Practice-Based Research Network (CEPBRN)* | *Practice-Based Projects* | *Clinicians, Patients, Investigators* | *Connect primary care practices and researchers to broaden research engagement* |
|  | *Community Advisory and Action Board (CAAB)* | *Infrastructure: Other* | *Community Members* | *Translate research from the Advance CTR into clinical public health practice and policy* |
|  | *Relationship Building* | *Relationship Building* | *Community Members, Investigators, Institutional Partners* | *Build relationships with community and institutional partners* |
|  | *Community Engagement Database* | *Sustainability Planning* | *CTR Leadership* | *Build a customer relationship management (CRM) database to track community activities and connect community needs with university resources* |
|  | *Training Sessions* | *Trainings* | *Investigators* | *Provide training to enhance community-engaged research efforts* |
| ***Alliance (Puerto Rico)*** | *Community Forums* | *Knowledge Transfer: Other* | *Community Members, Clinicians, Investigators* | *Identify health service needs among community members living in Puerto Rico* |
|  | *Symposium* | *Knowledge Transfer: Other* | *Community Members, Clinicians, Investigators* | *Discuss diagnostics, causes, risk factors, management, and other information related to a health need* |
|  | *Workshops* | *Trainings* | *Community Members, Clinicians, Investigators* | *Provide Community-Engaged Research (CEnR) training to a wide audience to build capacity and skill* |
|  | *Conferences* | *Knowledge Transfer: Other* | *Community Members, Clinicians, Investigators* | *Discuss the prevention and treatment of priority health concerns* |
|  | *Training Sessions* | *Trainings* | *Community Members, Clinicians, Investigators, Students* | *Provide training to enhance community-engaged research efforts* |
|  | *Community-Academic Partnership Support* | *Community-Academic Partnerships* | *Community Members, Investigators* | *Provide support for community members and investigators initiating and sustaining partnerships* |
|  | *Consultations* | *Consultations* | *Community Members, Investigators* | *Provide community-engaged support for community members and investigators throughout the research process* |
|  | *Pilot Grants* | *Pilot Grants* | *Community Members, Investigators* | *Provide community reviews and feedback on research pilot proposals* |
|  | *Community Health Research Advisory Council Meetings* | *Infrastructure: Other* | *Community Members* | *Develop a shared agenda, learning experiences, work plan, and provide community feedback on community-based projects* |
| ***Great Plains (Nebraska)*** | *Community-Engaged Research Institute* | *Trainings, Community-Academic Partnerships* | *Investigators, Community Partners* | *Provide bi-directional training on CE and CEnR principles, methodology, impact evaluation, and dissemination* |
|  | *Community-Academic Partnership Grant* | *Pilot Grants, Designing CEnR Projects in Partnership, Community-Academic Partnerships, Relationship Building* | *Investigators, Community Partners, Practice-Based Research Network* | *Provide pilot funding for CEnR projects across the network* |
|  | *Consultations* | *Consultations* | *Investigators, Community Partners* | *Provide support for CEnR in the form of recruitment, partnership initiation, methodology, grant support, and dissemination* |
|  | *Community-Engaged Research Interest Group* | *Knowledge Transfer: Other* | *Investigators, Community Partners* | *A forum on CEnR that creates space to discuss successes, roadblocks, and solutions for critical CEnR activities* |
|  | *Community Engagement Network* | *Financial/Budget/Incentive Concerns, IRB Approval for CEnR Projects, P&T Guidelines for CEnR, CE Leadership* | *CE Leadership* | *Institutional transformation for CEnR, update University policies and procedures to accommodate and enhance CEnR* |
|  | *Community Advisory Board* | *Relationship Building, Infrastructure: Other* | *Community Partners* | *Establish community-driven health priorities for research, increase community dissemination of research, and provide community feedback on grant proposals* |
| ***LA CaTS (Louisiana)*** | *Community Research for Optimal Wellness Network (CROWN)* | *Knowledge Transfer: Other, Trustworthiness, Relationship Building, Sustainability Planning* | *TBD* | *Develop infrastructure to enhance the reach and quality of LA CaTS’ community engagement, including sharing resources for health improvement/wellness, disseminating lay summaries of research results, and capacity building activities* |
|  | *Louisiana Community Scholars Program (LaCoSP)* | *Pilot Grants, Designing CEnR Projects in Partnership, Community-Academic Partnerships* | *Community Members, Investigators* | *Proposal Preparation Webinar Series, Kickoff Retreat, LaCoSP Seminar Series, and Personalized Mentorship Sessions* |
| ***MCCTR (Mississippi)*** | *CEnR Summer Institute* | *Trainings* | *Junior Investigators* | *Training designed to teach principles of CEnR* |
|  | *Digital Education Modules* | *Trainings* | *Investigators* | *Research Basics including CEnR* |
| ***Mountain West CTR (Montana, Wyoming, Idaho, Nevada, New Mexico, Alaska, and Hawaii)*** | *PBRN* | *Community-Academic Partnerships, Relationship Building, Infrastructure: Other* | *Community Members, Investigators, Clinicians* | *Build PBRN Infrastructure and develop guidelines for collaborative research initiatives* |
|  | *Training Programs* | *Trainings* | *Community Members, Investigators* | *Design and implement comprehensive education and training programs to promote research proficiency, cultural sensitivity, and high-quality research* |
|  | *Engage for Equity (E2) Activity Adaptation* | *Pilot Grants, Designing CEnR Projects in Partnership, Community-Academic Partnerships, Trustworthiness, Relationship Building* | *Community Members, Investigators* | *Adapt the Engage for Equity (E2) intervention developed by community-academic*  *partnerships in New Mexico to advance power-sharing*  *and equity using tools* |
|  | *Mentorship* | *Trainings* | *Community Members, Investigators, Staff, Students* | *Mentoring and training new investigators to help teams navigate the complex logistics of working with communities* |
|  | *Consultations* | *Consultations* | *Investigators* | *Provide guidance on community impact for intramural funding* |
| ***Montana CTRC (Montana)*** | *PBRN Development* | *Relationship Building, Infrastructure: Other* | *Community Members, Investigators, Clinicians* | *Bring community, PBRN, and research together to understand each other and identify specific research topics of interest* |
| ***NNE-CTR (Northern New England)*** | *Educational Presentations* | *Trainings, Community-Academic Partnerships* | *Investigators, Community Partners* | *Provide training on the basics of CEnR* |
|  | *Consultations* | *Consultations* | *Investigators* | *Discuss and provide support for CEnR basics, stakeholder readiness, evaluation, recruitment, budgeting, and grant writing* |
|  | *Community Engagement Studio* | *Consultations* | *Investigators, Community Partners* | *Provide education on CEnR, including methodology and levels of community engagement, and facilitate discussion between investigators and community members with lived experience* |
|  | *Regional Community Advisory Council* | *Designing CEnR Projects in Partnership, Infrastructure: Other* | *Investigators, Community Partners, Rural Clinicians, Stakeholders* | *Build long-term relationships with community partners and stakeholders* |
|  | *Boot Camp Translation Training* | *Trainings* | *Investigators, Research Staff, Community Partners* | *Prepare investigators and community partners to engage a group of 10-15 community members with lived experiences in a topical area to facilitate an iterative process to translate medical information into locally relevant language and messages to promote community action* |
|  | *Vermont Community Engagement Council* | *Infrastructure: Other* | *Community Partners, Vermont Health Department* | *Prioritize research topics in Vermont, strategize communicating with members of the community, and build partnerships* |
|  | *Vermont-Maine Bistate Community Engagement Council* | *Infrastructure: Other* | *Community Partners* | *Establish research priorities in Northern New England, build long-term partnerships* |
| ***OSCTR (Oklahoma)*** | *Practice-based research* | *Practice-Based Projects, Community-Academic Partnerships, Relationship Building* | *Clinicians, Patients, Investigators* | *Engage primary care practices and communities in practice-based research, education, learning collaboratives, and networking* |
|  | *Consultations* | *Consultations* | *Community Partner Organizations, Public Health Institute of Oklahoma* | *Provide technical assistance and staffing to support the design and organization of community-based research* |
|  | *Meetings and conferences* | *Trainings* | *Community Health Representatives* | *Engaging Tribal Nations through unifying community concerns and interests related to health care in rural and urban communities* |
|  | *Central Oklahoma Health Impact Team (COHIT)* | *Knowledge Transfer: Other* | *Health System Leaders* | *Conduct a community health needs assessment and community health improvement plan* |
| ***PIKO (Hawaii)*** | *Institute* | *Trainings* | *Community Members, Investigators* | *Networking opportunity, Lunch & Learn* |
|  | *Hawaii PCBRN* | *Practice-Based Projects* | *Community Health Centers and Organizations Leadership, Clinicians, Investigators* | *Build a robust practice and community research network for Hawaii by establishing relational agreements, protocols and policies for multi-site research and identifying priority topics* |
|  | *Community Advisory Board* | *Infrastructure: Other* | *Community Members at Center, Investigators* | *Guides the CEO Core, assists with community research assessments and reviews PIKO research protocols, enhances and assists with identifying and inviting members to the Laulima Network, a CTR research network, to link researchers with research-ready community partners by research foci and interests* |
| ***WVCTSI (West Virginia)*** | *Design Studio* | *Trainings, Consultations* | *Investigators, clinicians, community members* | *Didactic education session aimed to enhance methodology, research design, and data analysis* |
|  | *Ambassadors for Community Health Research (ACHR)* | *Trainings, Designing CEnR Projects in Partnership, Community-Academic Partnerships, Trustworthiness, Relationship Building, Sustainability Planning* | *Community Members* | *Provide training and education to better understand clinical and community research* |
|  | *West Virginia Area Health Education Centers (AHEC) Research Scholars Clinical and Translational Science Track* | *Trainings* | *Health Professional Students* | *Provide supplemental didactic and experiential learning opportunities to enhance skill sets and cultivate interest in engaging with rural and underserved West Virginia populations* |
|  | *Community Engagement and Outreach Community Forums* | *Designing CEnR Projects in Partnership, Community-Academic Partnerships, Knowledge Transfer: Other, Trustworthiness* | *Community Members* | *Promote bi-directional communication and match communities with researchers and implementation scientists who can partner for community-engaged solutions to identified health issues* |
|  | *WVCTSI Project Extensions for Community Healthcare Outcomes (ECHO)* | *Trainings* | *Clinicians, Community Health Workers, Administrators* | *Training and knowledge sharing to increase workforce capacity to provide best-practice specialty care and reduce health disparities* |
|  | *WVCTSI Community Advisory Board (CAB)* | *Trainings, Community-Academic Partnerships, Trustworthiness, Infrastructure: Other* | *Community Members* | *Provide leadership and direction to the WVCTSI by representing the community's interests and perspectives in the development, implementation, evaluation, dissemination, and administration of research projects* |
|  | *Implementation Science Center (ISC)* | *Trainings, Evidence-Based Projects, Practice-Based Projects, Community-Academic Partnerships, Sustainability Planning* | *Investigators, Clinicians, Community Members* | *Drive the uptake of research findings and improve health outcomes in rural West Virginia* |
|  | *Implementation Science Pilot Grant* | *Trainings, Pilot Grants, Community-Academic Partnerships* | *Investigators, Clinicians* | *Offer a pilot funding mechanism focused on community-based participatory research (CBPR)* |
|  | *Community Extensions for Community Healthcare Outcomes (ECHO)* | *Trainings, Practice-Based Projects, Trustworthiness, Sustainability Planning* | *Clinicians, Community Health Workers, Administrators* | *Expand the ECHO program to primary care sites to serve as the expert hub team, sharing steps taken to develop and complete processes, policies, and/or practice changes* |
